# Dynamic role and importance of surrogate species for assessing potential adverse environmental impacts of genetically engineered insect-resistant plants on non-target organisms

**DOI:** 10.1007/s11248-016-9945-5

**Published:** 2016-02-27

**Authors:** Michael Wach, Richard L. Hellmich, Raymond Layton, Jörg Romeis, Patricia G. Gadaleta

**Affiliations:** Center for Environmental Risk Assessment, ILSI Research Foundation, Washington, DC USA; USDA–ARS, Corn Insects and Crop Genetics Research Unit and Department of Entomology, Iowa State University, Ames, IA USA; DuPont Pioneer, Johnston, IA USA; Agroscope Reckenholz-Tänikon Research Station ART, Zurich, Switzerland; Biotechnology Directorate, Ministry of Agriculture, Livestock and Fisheries, Buenos Aires, Argentina

**Keywords:** Surrogate species, Genetically engineered insect resistance, Environmental risk assessment

## Abstract

Surrogate species have a long history of use in research and regulatory settings to understand the potentially harmful effects of toxic substances including pesticides. More recently, surrogate species have been used to evaluate the potential effects of proteins contained in genetically engineered insect resistant (GEIR) crops. Species commonly used in GEIR crop testing include beneficial organisms such as honeybees, arthropod predators, and parasitoids. The choice of appropriate surrogates is influenced by scientific factors such as the knowledge of the mode of action and the spectrum of activity as well as societal factors such as protection goals that assign value to certain ecosystem services such as pollination or pest control. The primary reasons for using surrogates include the inability to test all possible organisms, the restrictions on using certain organisms in testing (e.g., rare, threatened, or endangered species), and the ability to achieve greater sensitivity and statistical power by using laboratory testing of certain species. The acceptance of surrogate species data can allow results from one region to be applied or “transported” for use in another region. On the basis of over a decade of using surrogate species to evaluate potential effects of GEIR crops, it appears that the current surrogates have worked well to predict effects of GEIR crops that have been developed (Carstens et al. GM Crops Food 5:1–5, 2014), and it is expected that they should work well to predict effects of future GEIR crops based on similar technologies.

## Introduction

Surrogate species are those used to represent or substitute for other species. Surrogate species have a long history of use to evaluate potentially harmful effects of toxic substances. For example, beginning in the early 1900s mice and small birds were used in coal mines to detect carbon monoxide and thus improve worker safety (Acott et al. 1999; USDOL 2015). Other animal models came into use in laboratory testing after Trevan proposed comparing the toxicity of substances using LC50 values to predict effects on humans (Trevan 1927). Animal models, including various in vitro models, have been used for decades as surrogates for humans to assess the safety of pharmacology products and medical treatments. The first use of surrogates in ecological studies is attributed to Moore who conducted research on the environmental health of heath areas in the United Kingdom (Moore 1962). He used ten species (two plants, four insects, two lizards, and two birds) to evaluate the effect of land use changes. Over time various surrogate species (e.g., bobwhite quail, rainbow trout, water flea) began to be used successfully to provide environmental risk assessors with data to help make regulatory decisions regarding pesticides and other chemicals (Urban and Cook 1986).

Interest in protecting the ecological systems within agricultural systems began as a minor element of pest control in the 1940s, which then, during the late 1960s, developed into the practice of integrated pest management (IPM) with emphasis on preserving populations of beneficial insects (Ehler 2006). Testing systems for beneficial arthropod predators and parasites based on surrogates were developed (Croft and Strickler 1983; Hassan and Vogt 2006), however, there were no standardized protocols. In 1974 the International Organisation for Biological Control (IOBC) began the development of standardized tests for beneficial arthropods and introduced a tiered approach, with iterative tests, selection criteria for test species, and methods to interpret data (Hassan and Vogt 2006).

Genetically engineered insect resistant (GEIR) crops were developed in the 1990s and offered effective control over various insect pests. In 2014, GEIR crops were cultivated in 28 countries on 181 million ha worldwide (James 2014). Prior to cultivation in each country, these crops pass through a regulatory evaluation—including an environmental risk assessment. This paper provides a review of the use of surrogate species in the context of the environmental risk assessment (ERA) of GEIR crops. The first section provides an overview of the use of arthropod surrogates for the ERA of synthetic chemical pesticides and control agents. The second section describes how the existing methods using arthropod surrogates were applied to the evaluation of potential non-target effects of GEIR crops. Finally, the paper proposes ways to broaden the utility and transportability of environmental risk assessments informed by data from surrogate species testing in order to improve the risk assessment process, prevent the generation of duplicative data, and increase the consistency and efficiency of regulatory decision making.

## Surrogates in ERA for conventional pesticides

Most of the early efforts to test effects of pesticides on beneficial non-target arthropods were made in Europe. In 1974 the West Palaearctic Regional Section (WPRS) of the IOBC started to develop standardized tests for beneficial arthropods (Hassan and Vogt 2006). The motivation of the IOBC/WPRS efforts for establishing surrogates for non-target testing was to identify pesticides with limited adverse effects on beneficial arthropods under field conditions that would be compatible with IPM and integrated crop management (ICM) practices. The Pesticides and Beneficial Organisms Working Group of the IOBC/WPRS evaluated which surrogate species would be most useful and ultimately developed standard tests for nearly two dozen natural enemy species. The joint testing efforts initially focused on laboratory tests but then expanded to include semi-field and field procedures (Hassan et al. 1985, 1987; Croft 1990). The IOBC/WPRS efforts created a foundation for non-target arthropod (NTA) testing, and ERA requirements for European registration were defined in three international multi-stakeholder workshops: ESCORT 1 (European standard characteristics of non-target arthropod regulatory testing; (Barrett et al. 1994)), ESCORT 2 (Candolfi et al. 2001) and ESCORT 3 (Alix et al. 2012). Many of these protocols for evaluating non-target effects of arthropod control substances and have been used by regulators worldwide.

Standard procedures for regulatory testing of pesticides were agreed upon at the ESCORT 1 workshop. This included the application of a hierarchical, tiered testing scheme and the request of NTA data from four to six species including two species known to be particularly sensitive (a predatory mite, *Typhlodromus pyri* and an aphid parasitoid, *Aphidius rhopalosiphi*), and up to four crop-relevant species that are representative of ground- and foliage-dwelling predators and amenable to laboratory testing (Barrett et al. 1994). This marked the start of a systematic evaluation of the non-target effects of pesticides, and several standardized and ring-tested laboratory test methods were subsequently published (Candolfi et al. 2000). ESCORT 2 built upon ESCORT 1 and resulted in two major studies that evaluated the sensitivity of arthropods representing many NTA species across multiple orders to nearly 100 different pesticides (Candolfi et al. 1999; Heimbach et al. 2000). The species were selected based on commercial availability, amenability to testing in the laboratory, availability of validated test protocols, provision of sufficient phylogenetic and functional diversity, and representation of species that are present in agricultural fields and exposed to pesticides (Barrett et al. 1994; Candolfi et al. 2001). ESCORT 3 shifted the focus from in-crop risk assessment to off-crop areas, thus reaching the issue of biodiversity (Alix et al. 2012). One conclusion from the workshop was that the information and recommendations contained in current guidance documents produced during ESCORT 1 and 2 are applicable for conducting ERA for off-crop areas. In addition to the NTA guidance developed by the ESCORT workshops, many regulatory jurisdictions also require testing of honeybees. This is due to new pesticide regulations (European Commission 2009) based on biodiversity protection. Since the value of wild bees is of increasing interest in recent years, testing has been expanded to *Bombus* spp. and solitary bees (EFSA 2013). Similarly, surrogates that contribute to ecological functions in the soil have been proposed. For early tier studies in the laboratory these include the springtail *Folsomia candida*, the predatory mite *Hypoaspis aculeifer*, and dung beetles (Römbke 2006).

## Surrogates in ERA for GEIR

The successful development and use of NTA surrogates in predicting the potential effects of conventional pesticides in Europe has had a significant effect on the development of testing to evaluate potential environmental effects of GEIR crops. For example, an important criterion for selection of surrogate species was potential exposure in the field (Romeis et al. 2011). Surrogate species selected during the ESCORT process also could be used for testing associated with GEIR crops, although many of the tests developed during the ESCORT process were modified for use in testing pesticidal proteins in the laboratory: the ESCORT tests utilized contact or dermal exposure, GEIR testing required oral exposure. Criteria, such as availability of test organisms and protocols for testing and evaluating data were easily applied to selecting organisms for use in testing GEIRs. For example, test species commonly used for testing GEIR crops prior to cultivation approval in the United States include the earthworm and arthropod taxa including honeybees and three species of predators (from the orders Hemiptera, Coleoptera, usually ladybird beetles, Neuroptera, Hymenoptera, and Acarina) and parasitoids (from the orders of Diptera and Hymenoptera). The USEPA has recommended that testing should be performed on species from at least two of these groups; plus selection should take into account factors such as likelihood of exposure and phylogenetic relationship of test species to the target pest species (USEPA 1996). Phylogenetic relationships have been shown to be useful in the evaluation of possible adverse effects from insect resistance mediated by Bt proteins and double-stranded RNA (Romeis et al. 2009, 2013; Bachman et al. 2013). Lack of activity against a NTA surrogate species within the same order as the target species, such as Hymenoptera, provides assurance that species in more distantly related orders, such as Hemiptera, are also very unlikely to be affected. The need for testing is thereby reduced as the phylogenetic distance increases from the target spectrum. For exposure, lists of potentially exposed species will be similar to those already established for conventional pesticides.

In addition, knowledge about the mode of action of the compound and its spectrum of activity can inform the selection of species that are likely to be sensitive to the stressor of concern and thus provide the most rigorous test of the risk hypothesis. Other factors, such as the high value of certain ecosystem services, such as pollination by honeybees and decomposition by *Collembola* species (Romeis et al. 2013), also may help determine the selection of surrogates, even when there is no scientific reason to suspect harm from the stressor.

## Other considerations

The risk assessment that precedes the commercial use of genetically engineered crops is guided by broad environmental policies and protection goals, such as the protection of biodiversity and sustainable agricultural production (Wolt et al. 2010; Gray 2012; Garcia-Alonso and Raybould 2013). These policies and goals share common elements with those associated with the risk assessment of conventional pesticides. The selection of species data needed to evaluate the potential effects of GEIR crops requires the risk assessor to define the time period during which it should be protected, in order to translate broad environmental policies and protection goals into risk assessment operational goals (USEPA 2003; Gray 2012; Sanvido et al. 2012; Garcia-Alonso and Raybould 2013). This evaluation is done as part of problem formulation where, based on literature and inputs from experts, plausible links for both hazard and exposure are established between the stressor and protected entities.

There are three key reasons for using surrogates as part of the ERA process for GEIR crops. The first reason surrogates are used is the disruptive effect and cost of sampling and analysis: it is simply impossible to test and collect all species that are present in the receiving environment, and any attempt to do so would greatly disturb the agroecosystem and affect subsequent sampling. Thus, sampling methods are devised, and surrogate species are selected to represent the range of species potentially exposed to the particular environmental impact in question. The second reason surrogates are used is the case of assessing impacts to threatened or endangered species—even if these species could be reared in the laboratory, they are subject to certain legal restrictions. A third reason surrogates are used is that laboratory studies offer greater statistical power and endpoint sensitivity over field studies. Thus surrogate species are used to obtain information that can then be extrapolated to threatened and endangered species.

Given that it is impossible to test all non-target species potentially exposed to a control product, test species must be selected that represent the range of species potentially exposed to the pesticide (Raybould et al. 2011; Romeis et al. 2013). However, many organisms are not amenable to laboratory testing, usually because validated protocols and standardized diets are not available to rear and maintain consistent populations of organisms. Validated test protocols should be available for the species to ensure that the data obtained from the experiments are robust and reliable.

Tiered testing has been shown to be effective for identifying adverse direct effects on non-target organisms and establishing a lack of environmental harm arising from cultivation of GEIR crops, including those expressing Bt proteins (Duan et al. 2010). Extensive Tier I and Tier II studies suggest that, across many Bt transformation events and GEIR crop species, field studies are rarely, if ever, necessary to conclude a lack of ecologically relevant direct effects on NTA (Romeis et al. 2006; Marvier et al. 2007; Wolfenbarger et al. 2008; Naranjo 2009; Comas et al. 2014). The utility of early tier tests using surrogate species for conservatively predicting field effects has enabled regulatory agencies such as the USEPA to drop confirmatory field studies that were a condition of registrations during the first decade of commercial development of GEIR crops (USEPA 2014).

The use of surrogate species in tiered testing means that the results may be applicable, or transportable, to be used in risk assessments across countries and GEIR crops (Romeis et al. 2009; Raybould and Quemada 2010). This is particularly the case with early tier studies conducted under controlled conditions. The transportability of data from early-tier tests is greatly enhanced if the test methods are robust and designed to meet the quality standards of regulatory authorities in those jurisdictions where the genetically engineered insect-resistant event may be released for cultivation (Romeis et al. 2011). The Cartagena Protocol on Biodiversity encourages the use of any relevant scientific evidence that informs the risk assessment process, including evidence developed out of the country (CBD 2000), and in practice, the same eco-toxicology studies of a specific test substance could be reviewed by multiple competent authorities as part of pre-market ERAs. In situations where semi-field or field studies (sometimes referred to as Tier III and IV) are needed to provide critical data to refine the risk assessment, careful selection of experimental endpoints based on surrogate species used in early tier tests can facilitate data transportability. This can be relatively straightforward since most countries have protection goals that apply to a common set of valued ecological functions (e.g., pollination, biological control, etc.), and there is no scientific rationale to support the idea that NTA susceptibility is linked to political boundaries. Results from field experiments that directly measure these ecological functions can inform ERAs in the country where the study was conducted and also in other countries with similar receiving environments (Garcia-Alonso et al. 2014).

In addition to surrogate species, there are also other surrogate measures that can be applied. Direct measures of ecological functions are possible alternatives to field collections of arthropods. For example, methods are established to assess ecosystem services such as biological control, pollination, and decomposition of organic matter. Data from egg cards or sentinel hosts provide surrogate data for actual field effects (Luck et al. 1988). Seed set in potted plants has been used as a surrogate for pollination response (Jarlan et al. 1997). Decomposition of GE plant materials in litter bags has been used to provide surrogate data in both terrestrial and aquatic systems (Hönemann et al. 2008; Axelsson et al. 2010).

Surrogate species will continue to be used in future assessments of GEIR crops even as ERA processes change. An ecosystem services approach based on concepts in the Millennium Ecosystem Assessment (MEA) (Millenium Ecosystem Assessment 2005) has been proposed for the regulation of plant protection products (Nienstedt et al. 2012). Ecosystem services also have been proposed for use in ERA for GEIR crops by the European Food Safety Authority (Devos et al. 2015). Partitioning of common protection goals, such as biodiversity and sustainable agriculture, into ecosystem services helps define endpoints for risk assessment including: (1) entities to be protected from harm, (2) valued attributes of these entities (e.g., abundance or function), (3) unit of protection (individuals, populations, or functions), (4) spatial scale of protection (e.g., crop, non-agricultural habitats), and (5) temporal scale or protection (e.g., present or following cropping season) (Sanvido et al. 2012; Garcia-Alonso and Raybould 2013). Such an approach is useful to build links between regulated products and protected entities (i.e., defining pathways to harm) and to develop testable risk hypotheses (Gray 2012; Garcia-Alonso and Raybould 2013). Formulated risk hypotheses can then be tested within a tiered framework that moves from laboratory or early-tier tests, to more complex (higher tier) experiments, when necessary, which evaluate risks under more realistic exposure conditions (Hill and Sendashonga 2003; Garcia-Alonso et al. 2006; Romeis et al. 2008). The results from early tier testing are regarded as highly conservative, i.e., if an NTA is not affected under confined and controlled laboratory exposure conditions, the NTA is unlikely to be affected in the field. Through problem formulation and selection of appropriate surrogate species, the tiered testing process can be used to evaluate the potential effects on ecosystem services.

## Conclusions and recommendations

For over 50 years surrogate species have been used extensively to assess the effects of environmental stressors on various organisms. Although surrogate species testing may have originally been adopted for the simple reason that not all non-target organisms could be tested, the value of surrogate species in environmental risk assessment has been recognized globally and is now standard practice for the generation of ERA data. This is because surrogate species testing can generate consistent data, of high statistical power, that accurately predicts the environmental impacts of a given stressor. Data regarding impacts from GEIR crops on surrogate species are informing regulatory decision making in every country that has considered the commercial deployment of these crops, and the track record of safe use of GE crops demonstrates the value and utility of surrogate species tests.

However, the fact remains that there continues to be disharmony among national regulatory systems, resulting in needless duplication of environmental safety testing and worse, the generation of incongruent conclusions regarding the safety of GEIR crops. Given the volume of NTO effects data generated through the use of surrogate species and the depth of analysis to which these data have been subjected, the following conclusions support the transportability and the acceptance of these available data, as well as new data to be collected, using surrogate species for the ERA of new GEIR.

1. Current surrogates have worked well, based on a review of surrogate species tests and their ability to predict field level effects.
2. The surrogate species approach also should work well for newly developed GEIR using Bt proteins.
3. There does not appear to be a need for countries to perform NTA assessments on novel, local species simply because they are local, if an appropriate surrogate has already been tested.
4. Standards/criteria/protocols for laboratory testing using existing and newly identified surrogate species should be developed, validated, disseminated and used so that results are transportable.
